# Reply: Optimum duration of breast cancer follow-up: a continuing controversy

**DOI:** 10.1038/sj.bjc.6603974

**Published:** 2007-09-11

**Authors:** D A Montgomery, J M Dixon

**Affiliations:** 1 Glasgow Royal Infirmary, University Department of Surgery, Level 2, Queen Elizabeth Building, Glasgow G31 2ER, UK

**Sir**,

This is in response to the letter from Maraqa and Lansdown.

Our recent study was an attempt to present pattern of relapse, method of detection of that relapse and its impact on outcome in greater detail than has previously been reported in the literature.

For ipsilateral breast relapse, those clinically detected have a significantly poorer outcome than those detected by patients themselves or by mammography. As we stated, there is no difference in outcome between methods of detection for axillary relapse or contralateral cancers. All sites of relapse were analysed separately as we felt this highlighted the observation that more axillary than breast relapses are detected by clinicians. We mentioned that there should be more emphasis on educating patients to perform axillary self-examination.

Maraqa and Lansdown state that we reported clinically detected cancers do less well, although the relevant paragraph relates to ipsilateral breast relapses. It is clear from our Results section that this is not true for all sites of relapse. Where all relapses are discussed, we stated ‘our data suggest that regular clinical examination does not improve outcome’.

Figures 4 and 5 in our paper present data only on ipsilateral breast relapses; contralateral and axillary data were not excluded. As stated, when analysed separately, there was no difference between the three methods of detection for either site of relapse. If all sites of relapse are analysed together, as Maraqa and Lansdown correctly point out, there is no difference between methods of detection of relapse (Figure 1 below; log rank 2 d.f., *P*=0.0971), although this was not presented in our paper.

We feel that Maraqa and Lansdown are missing out the main point of our paper. The question is not whether palpable relapse does less well than mammographically detected relapse. An analysis of all patients including those with axillary relapse reveals that patients with palpable relapse do significantly less well than those with mammographically detected relapse (Figure 2; log rank 1 d.f., *P*=0.0325), as shown by others previously (Voogd, 1999).

The question is whether patients obtain any significant clinical benefit from attending routine clinics for clinical examination. In the past, studies have grouped all palpable relapses together or have combined all recurrences detected as part of follow-up. What is relevant is whether clinical examination contributes significantly to the detection of relapse, and what is the outcome of patients with palpable cancers who do not notice them on their own (ie, all patients with clinically diagnosed relapse).

In our study, very few relapses were detected clinically. Ipsilateral breast relapses found on clinical examination did less well. Clinical detection of contralateral breast relapse was very rare, with 2 events from 1312 patients over 10 years. Clinically detected axillary relapse was somewhat more common, 9 axillary relapses detected by clinical examination in 1312 women over 10 years of follow-up.

Potentially treatable relapse occurs at a constant rate, possibly for life. Clinical detection of such relapse is a rare event, at around 0.1–0.2% of women per year. Tremendous effort is currently expended in follow-up clinics for a very small yield, despite no benefit in outcome, and a poorer outcome in some types of relapse.

## Figures and Tables

**Figure 1:**
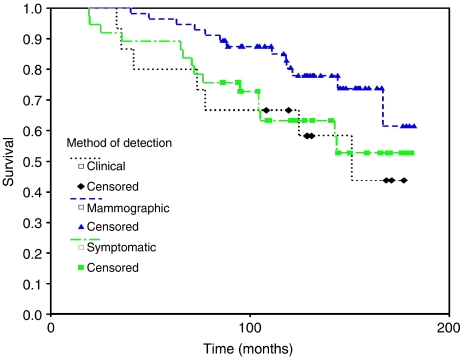
Overall survival for all sites of relapse (ipsilateral breast, axillary and contralateral breast) analysed by the method of detection.

**Figure 2:**
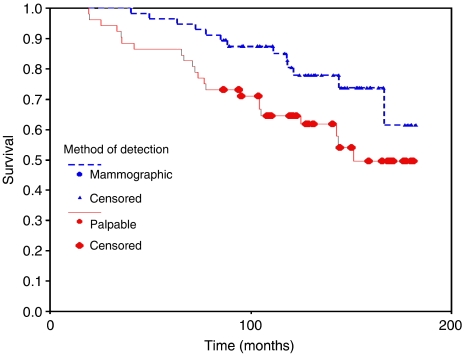
Overall survival for all sites of relapse (ipsilateral breast, axillary and contralateral breast) separated into palpable (clinician or patient detected) *vs* mammographically detected.
